# Differential Expression of Specific Dermatan Sulfate Domains in Renal Pathology

**DOI:** 10.1371/journal.pone.0134946

**Published:** 2015-08-31

**Authors:** Joost F. M. Lensen, Johan van der Vlag, Elly M. M. Versteeg, Jack F. M. Wetzels, Lambert P. W. J. van den Heuvel, Jo H. M. Berden, Toin H. van Kuppevelt, Angelique L. W. M. M. Rops

**Affiliations:** 1 Department of Biochemistry, Radboud Institute for Molecular Life Sciences, Radboud University Medical Center, Nijmegen, The Netherlands; 2 Nephrology Research Laboratory, Radboud Institute for Molecular Life Sciences, Department of Nephrology, Radboud University Medical Center, Nijmegen, The Netherlands; 3 Department of Pediatrics, Radboud University Medical Center, Nijmegen, The Netherlands; Fondazione IRCCS Ospedale Maggiore Policlinico & Fondazione D’Amico per la Ricerca sulle Malattie Renali, ITALY

## Abstract

Dermatan sulfate (DS), also known as chondroitin sulfate (CS)-B, is a member of the linear polysaccharides called glycosaminoglycans (GAGs). The expression of CS/DS and DS proteoglycans is increased in several fibrotic renal diseases, including interstitial fibrosis, diabetic nephropathy, mesangial sclerosis and nephrosclerosis. Little, however, is known about structural alterations in DS in renal diseases. The aim of this study was to evaluate the renal expression of two different DS domains in renal transplant rejection and glomerular pathologies. DS expression was evaluated in normal renal tissue and in kidney biopsies obtained from patients with acute interstitial or vascular renal allograft rejection, patients with interstitial fibrosis and tubular atrophy (IF/TA), and from patients with focal segmental glomerulosclerosis (FSGS), membranous glomerulopathy (MGP) or systemic lupus erythematosus (SLE), using our unique specific anti-DS antibodies LKN1 and GD3A12. Expression of the 4/2,4-di-O-sulfated DS domain recognized by antibody LKN1 was decreased in the interstitium of transplant kidneys with IF/TA, which was accompanied by an increased expression of type I collagen, decorin and transforming growth factor beta (TGF-β), while its expression was increased in the interstitium in FSGS, MGP and SLE. Importantly, all patients showed glomerular LKN1 staining in contrast to the controls. Expression of the IdoA-Gal-NAc4SDS domain recognized by GD3A12 was similar in controls and patients. Our data suggest a role for the DS domain recognized by antibody LKN1 in renal diseases with early fibrosis. Further research is required to delineate the exact role of different DS domains in renal fibrosis.

## Introduction

Dermatan sulfate (DS) is a member of the large family of linear polysaccharides called glycosaminoglycans (GAGs). DS is also known as chondroitin sulfate B (CS-B) and is composed of repeating disaccharide units consisting of N-acetyl galactosamine (GalNAc) and glucuronic acid (GlcA) residues. The presence of the epimerized form of GlcA, iduronic acid (IdoA), defines it as DS. Covalently bound to a core protein DS forms proteoglycans (PGs), such as decorin and biglycan [1, 2]. The level of complexity of DS is dictated by a variable structure and chain length of up to 40–100 disaccharide units. The uronic acid can be either a GlcA or IdoA with or without 2-O-sulfation, and the GalNAc residue can be 4- and/or 6-O-sulfated. These possibilities can result in the formation of several defined disaccharide units; CSA (4-O-sulfated), CSB/DS (IdoA and 2,4-di-O-sulfated), CSC (6-O-sulfated), CSD (2,6-di-O-sulfated), CSE (4,6-di-O-sulfated) and CSEi (IdoA and 4,6-di-O-sulfated) [3].

DS is able to bind a myriad of factors, including fibroblast growth factor (FGF)-1, -2 &-7, heparin cofactor II and interferon (IFN)-γ. Specific sulfation patterns and the amount of epimerization within a CS/DS chain dictate growth factor/cytokine binding and function, such as cell proliferation, signal transduction and extracellular matrix (ECM) modulation [2]. The biological roles of both the proteoglycan core protein and the DS side chains have been studied, mostly in relation to extracellular matrix components, especially collagens. These studies have indicated fundamental roles of the DSPGs biglycan and decorin in regulating collagen fibril formation *in vivo* [4, 5], while the DS side chains have been implicated in influencing the mechanical strength of collagen fibrils *in vitro* as well [6]. The increase in extracellular matrix and collagen is related to fibrosis and sclerosis.

Glomerulosclerosis and tubulointerstitial fibrosis are common final pathological features of most end-stage kidney diseases irrespective of the underlying etiology [7]. It is also the final common pathway of renal allograft damage, where the specific features are interstitial fibrosis and tubular atrophy, which can lead to glomerulosclerosis resulting in a decline in glomerular filtration rate [8]. Renal allograft injury can roughly be divided as acute, mediated by cellular and/or antibody mediated rejection, or as chronic with moderate to severe interstitial fibrosis, tubular atrophy and absence of specific glomerular pathology in an early stage (IF/TA) [9]. Tubulointerstitial fibrosis involves inflammation, proliferation, apoptosis and fibrosis. An early identification of renal allograft loss could improve long-term allograft survival. Both acute and chronic rejection are associated with increased chemokine expression, many of which are GAG-binding [10]. There is an increase in circulating GAGs in patients with acute allograft rejection [11] and experimental heparin/synthetic sulfated oligosaccharides therapy has been shown to prolong transplant function and reduce rejection [12, 13]. It was shown that the heparan sulfate (HS) PG expression was increased in sclerotic glomeruli, while the CSPG expression was increased in the fibrotic interstitium in a renal transplant model in rats [10]. CS/DS and DSPGs have been reported to be increased in several fibrotic renal diseases, including interstitial fibrosis, diabetic nephropathy, mesangial sclerosis and nephrosclerosis [14, 15]. Changes in CS/DS content and modifications have been found in different animal models for renal disease [14, 16–18]. However, the precise role of DS in the ECM of both the normal and the fibrotic kidney is not well understood. Importantly, little is known about structural alterations in DS in renal diseases due to lack of proper tools for detection. In particular, specific antibodies directed against specific domains in DS could further facilitate research on the role of DS in renal fibrosis. Some antibodies recognizing both DS and CS have been described [19], and previously we reported two specific anti-DS antibodies (GD3A12 and LKN1). The phage displayed-derived anti-DS antibody LKN1 was shown to be especially reactive with 4/2,4-di-O-sulfated DS, whereas antibody GD3A12 recognized IdoA-Gal-NAc4S [20, 21].

In the current study we elucidate the expression of the different DS domains defined by antibodies LKN1 and GD3A12 using a panel of renal biopsies obtained from patients with acute interstitial, acute vascular and chronic renal allograft rejections, and from patients with focal segmental glomerulosclerosis (FSGS), membranous glomerulopathy (MGP) and systemic lupus erythematosus (SLE). The expression of the DSPG decorin, type I collagen and transforming growth factor beta (TGF-β) was investigated as well. Our data revealed that the 4/2,4-di-O-sulfated DS domain recognized by antibody LKN1 was differentially expressed in patients, while expression of the IdoA-Gal-NAc4SDS domain recognized by GD3A12 was similar in controls and patients.

## Materials and Methods

### Human kidney specimens

This study was performed in accordance with the applicable rules concerning the review of the research ethics committee of the Radboud university medical center Nijmegen. We used archived renal biopsy material that was anonymized prior to us receiving these materials. Renal allograft biopsies were obtained from patients with (according to Banff ‘97 guidelines [22]) acute interstitial (n = 6; Banff’97 class I, C4d negative), acute humoral/vascular (n = 6; Banff’97 class II, C4d positive) and chronic (n = 5; with slight to moderate fibrosis and tubular atrophy, class II) rejection, later called “Interstitial fibrosis and tubular atrophy (IF/TA), without evidence of any specific etiology” [23]. Note that these biopsies were previously obtained and scored according to the Banff ‘97 guidelines. Needle renal biopsies were obtained from patients with focal segmental glomerulosclerosis (FSGS, n = 11), membranous glomerulopathy (MGP, n = 6), systemic lupus erythematosus (SLE, n = 6). Four renal biopsies not showing any signs of rejection or other pathology were used as controls.

### Immunofluorescence staining

Indirect immunofluorescence staining was performed on 2 μm cryostat renal sections. Sections were fixed in ice-cold acetone for 10 minutes and incubated with primary antibodies diluted in phosphate-buffered saline (PBS) containing 1% (w/v) bovine serum albumin (BSA, Sigma-Aldrich Chemie BV, Zwijndrecht, The Netherlands) and 0.05% sodium azide (IF-buffer) for 45 minutes at room temperature (RT). Primary antibodies included rabbit anti-bovine collagen type 1 (Millipore BV, Amsterdam, The Netherlands), mouse anti-human decorin (R&D Systems Europe Ltd. Oxon, United Kingdom), rabbit anti-transforming growth factor beta1 (TGF-β1) (Abcam, Cambridge, United Kingdom) and the VSV-tagged anti-DS antibodies LKN1 [20] and GD3A12 [21]. Sections were rinsed with PBS and incubated with anti-rabbit or anti-mouse Alexa 488-conjugated IgG (5 μg/ml, Molecular Probes, Leiden, The Netherlands) or anti-VSV-Cy3 (2 μg/ml, Sigma-Aldrich Chemie) in IF buffer for 45 minutes at RT. Sections were rinsed with PBS and subsequently fixed in 100% ethanol for 20 seconds, air-dried and embedded in Mowiol. The staining intensities of all antibodies were scored using arbitrary units (AU) on a scale between 0 and 4 (0 = no staining, 2 = intermediate staining and 4 = strong staining) by two investigators on blinded sections.

### Statistical analysis

Data are presented as mean ± standard deviation (s.d.) and, if not normally distributed, as median with interquartile ranges. For comparisons of different groups Mann-Whitney U tests were used. Statistical analysis was performed using GraphPad Prism 5.0 (GraphPad Software Inc., San Diego, CA, USA).

## Results

### Decreased expression of the 4/2,4-di-O-sulfated DS domain defined by antibody LKN1 in the tubular interstitium in interstitial fibrosis and tubular atrophy (IF/TA)

**Fig 1:**
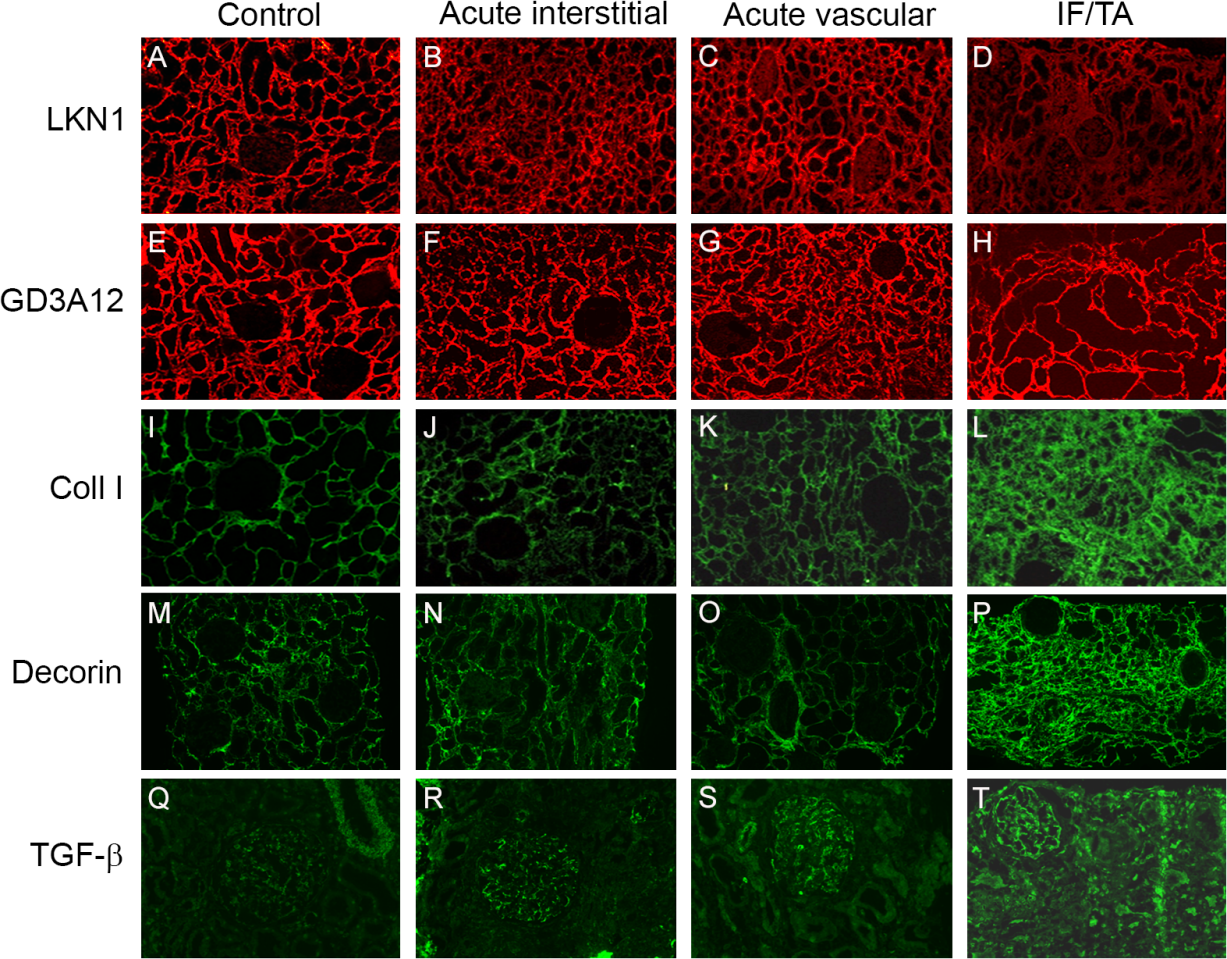
Expression of the 4/2,4-di-O-sulfated and IdoA-Gal-NAc4S DS domains defined by the antibodies LKN1 (A-D) and GD3A12 (E-H), type I collagen (I-L), decorin (M-P) and TGF-β (Q-T) in renal allograft rejection and controls. Representative photographs showing the expression of the 4/2,4-di-O-sulfated DS domain defined by LKN1 in the control human kidneys (A). Tubular interstitial expression of this 4/2,4-di-O-sulfated DS domain defined by LKN1 was increased in acute interstitial (B) and acute vascular (C) renal allograft rejections compared to interstitial fibrosis and tubular atrophy (IF/TA) (D). Expression of the IdoA-Gal-NAc4S DS domain recognized by GD3A12 was similar in the control human kidney and the three types of renal allograft rejection (E-H), while expression of type I collagen (coll I) and decorin was increased in IF/TA (L, P) compared to the control human kidney (I, M), and acute interstitial (J, N) and acute vascular renal allograft rejections (K, O). Glomerular expression of transforming growth factor beta (TGF-β) was increased in the three types of renal allograft rejection (R-T) compared to the control human kidney (Q). Magnification A-P 100x, magnification Q-T 200x.

**Fig 2:**
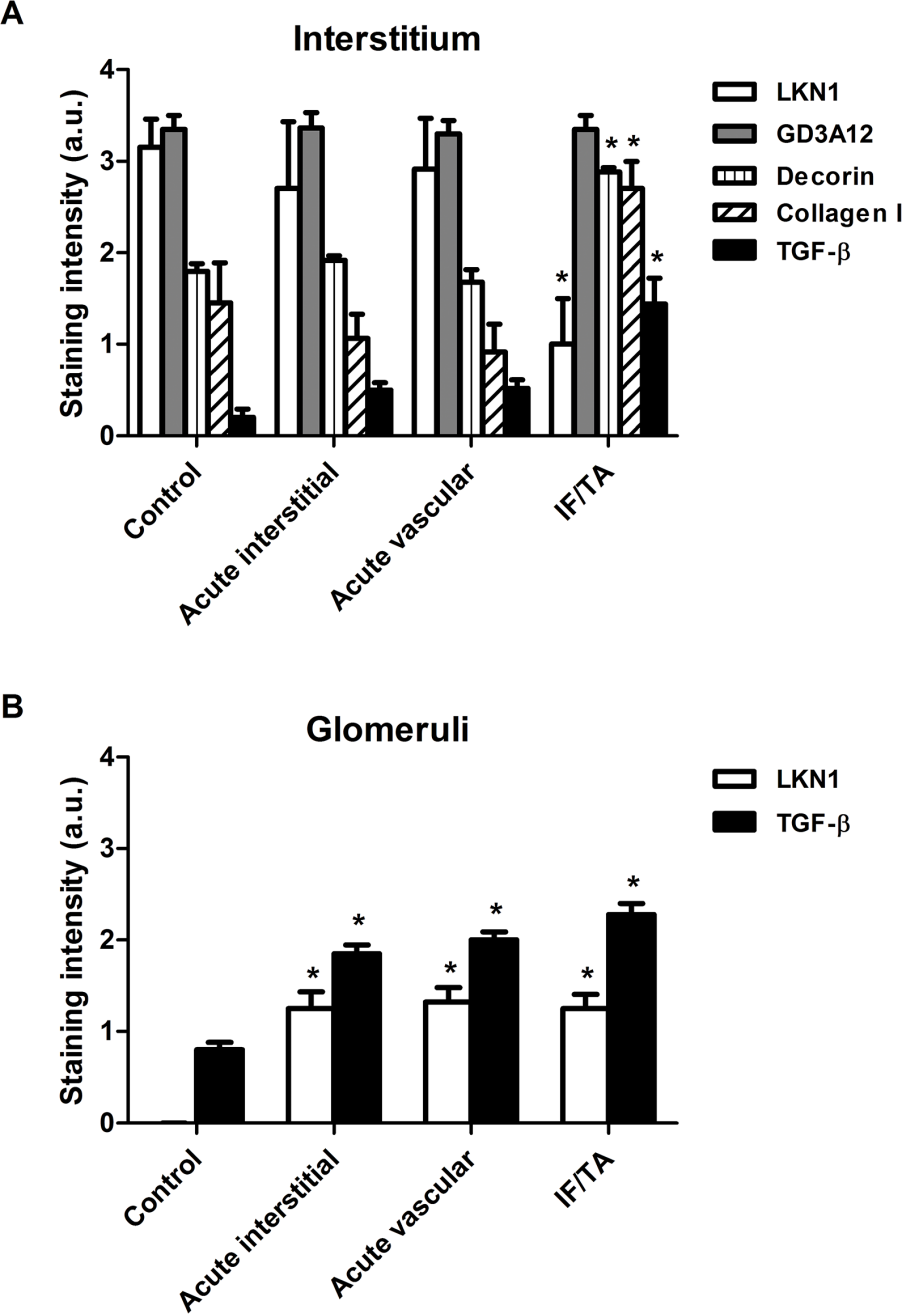
Semi-quantitative analysis of the expression of type I collagen, decorin, TGF-β and the 4/2,4-di-O-sulfated and IdoA-Gal-NAc4S DS domains defined by LKN1 and GD3A12 in the interstitium (A) and glomeruli (B) in renal allograft rejection. Staining intensities of type I collagen, decorin, transforming growth factor beta (TGF-β) and the DS domains defined by LKN1 and GD3A12 were scored using a scale of 0–4 and revealed a significantly increased tubular interstitial expression of type I collagen, decorin and TGF-β and a decreased expression of the 4/2,4-di-O-sulfated DS domain recognized by LKN1 in interstitial fibrosis and tubular atrophy (IF/TA). Expression of the IdoA-Gal-NAc4S DS domain recognized by GD3A12 was similar in controls and the three types of renal allograft rejection (A). Glomerular expression of the 4/2,4-di-O-sulfated DS domain defined recognized by LKN1 and TGF-β was increased in the acute interstitial, acute vascular and chronic renal allograft rejections compared to controls (B). *P<0.05 vs control.

The distribution of the 4/2,4-di-O-sulfated and IdoA-Gal-NAc4S DS domains recognized by, respectively, the LKN1 and GD3A12 antibody was analysed in biopsies with renal allograft rejection and controls (Figs 1 and 2). Relevant clinical data of the patients are given in Table 1. In controls, the 4/2,4-di-O-sulfated DS domain defined by LKN1 was expressed in the tubular interstitium, including the matrix surrounding Bowman’s capsule, with a distinct fibrillar expression pattern (Fig 1A). No expression inside the glomerulus was seen. Previously, we showed that this DS domain co-localized with type I collagen [20].

**Table 1:** Clinical characteristics of transplanted patients with rejection.

Patient	Sex	Age	Original kidney disease	Banff’97	Graft survival at time of biopsy	SCreat (μmol/l) at biopsy	Proteinuria (g/24h) at biopsy	SCreat (μmol/l) at last follow-up
**1**	**F**	**27**	**Tubulo-interstitial nephritis**	**I**	**10 months**	**170**	**0.4**	**Failure at 81 months**
**2**	**M**	**41**	**Infravesical obstruction**	**I**	**8 days**	**247**	**3.7**	**150 at 72 months**
**3**	**F**	**39**	**Glomerulonephritis and nephritic syndrome**	**Ia**	**16 months**	**107**	**0.25**	**99 at 108 months**
**4**	**M**	**39**	**ESRD unknown cause**	**Ib**	**9 months**	**171**	**0.72**	**229 at 87 months**
**5**	**M**	**15**	**MPGN type I**	**Ib**	**2 months**	**136**	**0.1**	**139 at 60 months**
**6**	**M**	**47**	**IgA nephropathy**	**I**	**5 months**	**162**	**1.37**	**206 at 72 months**
**7**	**M**	**22**	**Hypoplasia**	**Chronic**	**20 months**	**157**	**3.94**	**Failure at 23 months**
**8**	**M**	**40**	**ESRD unknown cause**	**Chronic**	**16 months**	**154**	**3.82**	**229 at 87 months**
**9**	**M**	**21**	**Urethral valves**	**Chronic**	**103 months**	**197**	**1.77**	**275 at 174 months**
**10**	**M**	**31**	**Urethral valves**	**Chronic**	**202 months**	**900**	**N.A.**	**Failure at 151 months**
**11**	**M**	**47**	**IgA nephropathy**	**Chronic**	**5 months**	**162**	**0.83**	**199 at 44 months**
**12**	**M**	**27**	**IgA nephropathy**	**IIa**	**9 months**	**149**	**0.43**	**143 at 84 months**
**13**	**M**	**66**	**Hemolytic uremic syndrome**	**IIa**	**1 months**	**308**	**0.58**	**Failure at 1 month**
**14**	**M**	**31**	**Medullary cystic disease**	**IIa**	**1 month**	**193**	**0.61**	**Failure at 25 months**
**15**	**M**	**53**	**ANCA-vasculitis**	**IIa**	**9 months**	**152**	**0.43**	**337 at 81 months**
**16**	**F**	**43**	**Chronic pyelonephritis**	**IIa**	**1 week**	**116**	**0.4**	**137 at 61 months**
**17**	**M**	**4**	**Urethral valves**	**IIa**	**1 month**	**83**	**0**	**69 at 71 months**

Abbreviations: SCreat, Serum creatinine; ESRD, End Stage Renal Disease; MPGN, Membranoproliferative glomerulonephritis; ANCA-vasculitis, Anti-neutrophil cytoplasmic antibodies-associated vasculitis; N.A., not available.

In IF/TA, expression of the 4/2,4-di-O-sulfated DS domain recognized by antibody LKN1 was significantly decreased in the tubular interstitium (Fig 1D), whereas the expression in the acute interstitial (Fig 1B) and vascular renal allograft rejection (Fig 1C) was similar to the control kidneys (Figs 1A and 2A). The strongly decreased expression of the 4/2,4-di-O-sulfated DS domain was accompanied by an increased expression of type I collagen, the DSPG decorin and TGF-β in IF/TA (Figs 1L, 1P, 1T and 2A). Type I collagen and decorin expression in acute interstitial (Fig 1J and 1N) and acute vascular renal allograft rejection (Fig 1K and 1O) was similar to the control kidneys (Fig 1I and 1M). Furthermore, glomerular staining of LKN1 was increased in IF/TA. and a similar increase also occurred in acute interstitial and vascular rejection (Fig 2B). Importantly, glomerular staining of TGF-β was also increased in acute interstitial and vascular rejection, and in IF/TA (Figs 1R–1T and 2B) compared to the control kidneys (Figs 1Q and 2B).

The expression pattern of the IdoA-Gal-NAc4S DS domain recognized by the GD3A12 antibody in control kidney was similar to the expression pattern of the 4/2,4-di-O-sulfated DS domain defined by LKN1. Thus, also in this case no glomerular expression was observed (Fig 1E). Furthermore, no differences were observed between the expression of this IdoA-Gal-NAc4SDS domain in the control group (Fig 1E), and in the groups of patients with acute interstitial (Fig 1F), acute vascular renal allograft rejection (Fig 1G) or IF/TA (Figs 1H and 2A).

### Increased expression of the 4/2,4-di-O-sulfated DS domain defined by antibody LKN1 in the tubular interstitium and glomeruli in human glomerular diseases

**Fig 3:**
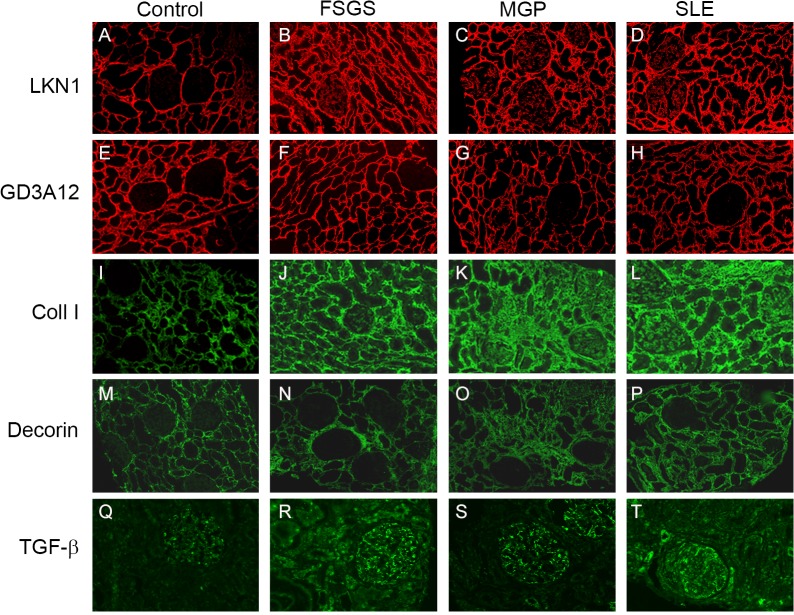
Expression of the 4/2,4-di-O-sulfated and IdoA-Gal-NAc4S DS domains defined by LKN1 (A-D) and GD3A12 (E-H), type I collagen (I-L), decorin (M-P) and TGF-β (Q-T) in renal sections obtained from patients with glomerular diseases. Representative photographs showing the immunofluorescence stainings of renal sections obtained from controls (A, E, I, M, Q), patients with focal segmental glomerulosclerosis (FSGS) (B, F, J, N, R), membranous glomerulopathy (MGP) (C, G, K, O, S) and systemic lupus erythematosus (SLE) (D, H, L, P, T) with the anti-DS antibodies LKN1 (A-D) and GD3A12 (E-H), anti-collagen type I (Coll I) (I-L), decorin (M-P) and transforming growth factor beta (TGF-β) (Q-T). Tubular interstitial and glomerular expression of the 4/2,4-di-O-sulfated DS domain defined by LKN1 and of type I collagen was increased in FSGS (B, J), MGP (C, K) and SLE (D, L) compared to control human kidney (A, I). Expression of the IdoA-Gal-NAc4S DS domain, recognized by GD3A12, and decorin was similar in the interstitium and glomeruli of patients (F-H, N-P) and controls (E, M). Glomerular expression of TGF-β was increased in FSGS (R), MGP (S) and SLE (T) compared to control (Q). Magnification A-P 100x, magnification Q-T 200x.

**Fig 4:**
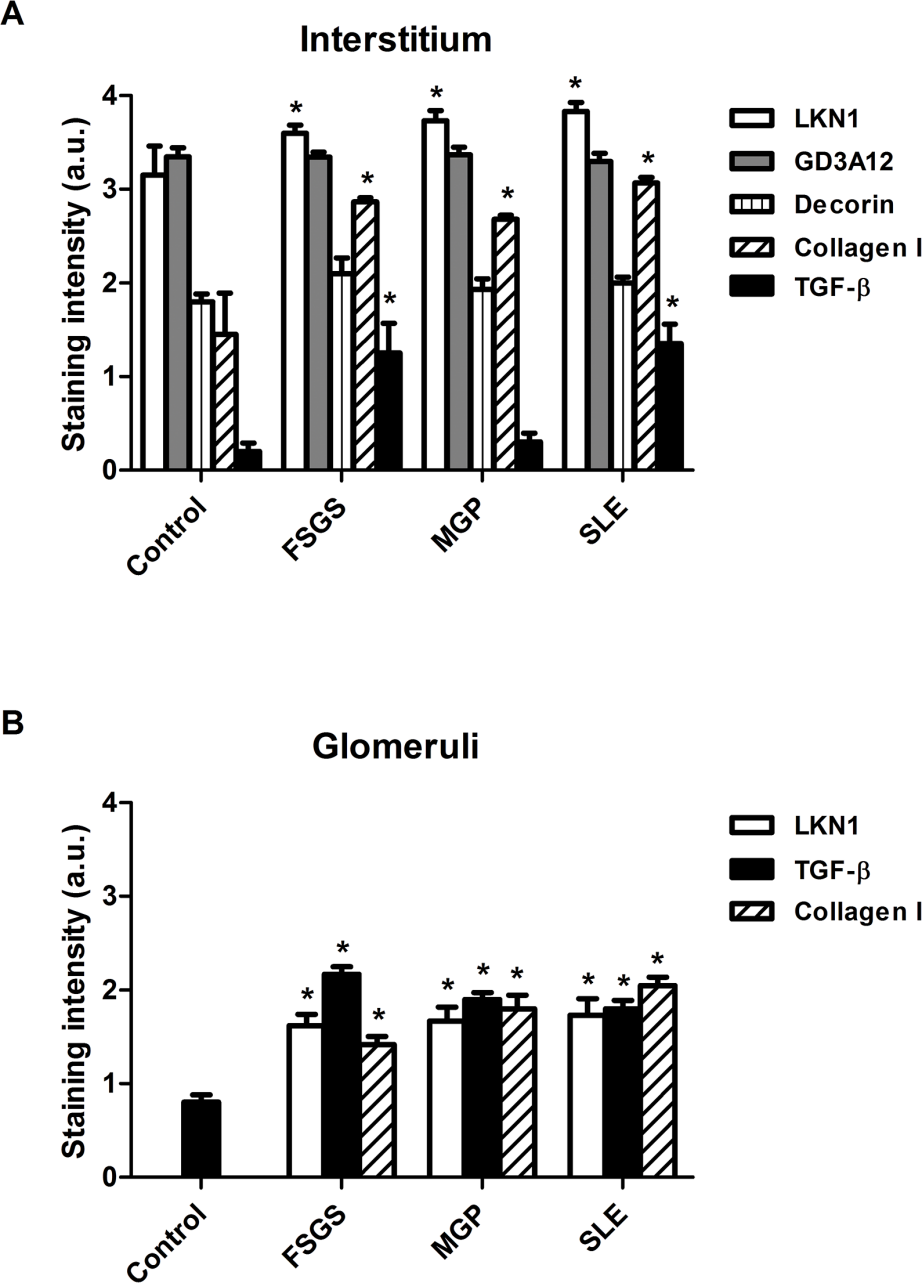
Semi-quantitative analysis of the expression the 4/2,4-di-O-sulfated and IdoA-Gal-NAc4S DS domains defined by LKN1 and GD3A12, type I collagen, decorin and TGF-β in the interstitium (A) and glomeruli (B) of patients with FSGS, MGP and SLE. Staining intensities of the 4/2,4-di-O-sulfated and IdoA-Gal-NAc4S DS domains defined by LKN1 and GD3A12 respectively and of type I collagen, decorin and transforming growth factor beta (TGF-β) were scored using a scale of 0–4 and revealed a significantly increased expression of the 4/2,4-di-O-sulfated DS domain and type I collagen in the interstitium (A) and glomeruli (B) of patients with glomerular diseases. Glomerular expression of TGF-β also was increased in patients with glomerular diseases (B), while expression of the IdoA-Gal-NAc4S DS domain and decorin was similar in the interstitium (A) of patients and controls. *P<0.05 vs control.

Since these 2 specific DS domains were differentially expressed in the different types of renal allograft rejection, we also analyzed their renal expression in three glomerular diseases FSGS, MGP and SLE. Relevant clinical data of these patients are given in Table 2. In all these glomerular diseases, the 4/2,4-di-O-sulfated DS domain, recognized by the LKN1 antibody (Fig 3B–3D), and type I collagen (Fig 3J–3L) were expressed in the glomerulus in contrast to the controls (Figs 3A, 3I, and 4B). Furthermore, expression of this DS domain was significantly increased in the tubular interstitium of patients with FSGS, MGP and SLE (Figs 3B–3D and 4A), which was accompanied by an increased expression of type I collagen as well (Figs 3J–3L and 4A). Expression of TGF-β was increased in the tubular interstitium of patients with FSGS and SLE (Figs 3R, 3T, and 4A). Importantly, the increased glomerular expression of the 4/2,4-di-O-sulfated DS domain was accompanied by an increased glomerular expression of TGF-β (Figs 3R–3T and 4B) compared to the controls (Figs 3Q and 4B). In contrast to the 4/2,4-di-O-sulfated DS domain, the IdoA-Gal-NAc4S DS domain, recognized by the antibody GD3A12, and decorin were not expressed in the glomeruli of patients with glomerular diseases (Fig 3F–3H and 3N–3P). Furthermore, the expression of the IdoA-Gal-NAc4S DS domain and decorin in the tubular compartment was similar in both normal and glomerular diseased kidneys (Figs 3E–3H, 3M–3P and 4A).

**Table 2:** Characteristics of patients with glomerular diseases.

	FSGS	MGP	SLE*
**N**	**11**	**6**	**6**
**Male (N)**	**9**	**4**	**0**
**Age (years)**	**54.3 (20–68)**	**55.2 (33–70)**	**25.8 (19–74)**
**Endogenous creatinine clearance (ml/min)**	**107 (24–163)**	**104 (30–153)**	**92 (36–136)**
**Serum albumin (g/l)**	**25 (14–42)**	**21 (12–24)**	**31 (24–45)**
**Proteinuria (g/day)**	**9.1 (5–15)**	**8.1 (2.5–15)**	**2.9 (0.8–15)**
**Glomerulosclerosis (0–3)**	**1 (0–3)**	**1 (0–1)**	**0 (0–2)**
**Interstitial fibrosis and tubular atrophy (0–3)**	**1 (0–3)**	**0.5 (0–3)**	**1 (1–2)**

Values are given as median and range. Abbreviations: FSGS, focal segmental glomerulosclerosis; MGP, membranous glomerulopathy; SLE, systemic lupus erythematosus. Glomerulosclerosis, interstitial fibrosis and tubular atrophy were scored as absent (0), < 25% (1), 25–50% (2), > 50% (3).

*All SLE patients had proliferative lupus nephritis (ISN/RPS class III: N = 3; class IV: N = 3).

**Fig 5:**
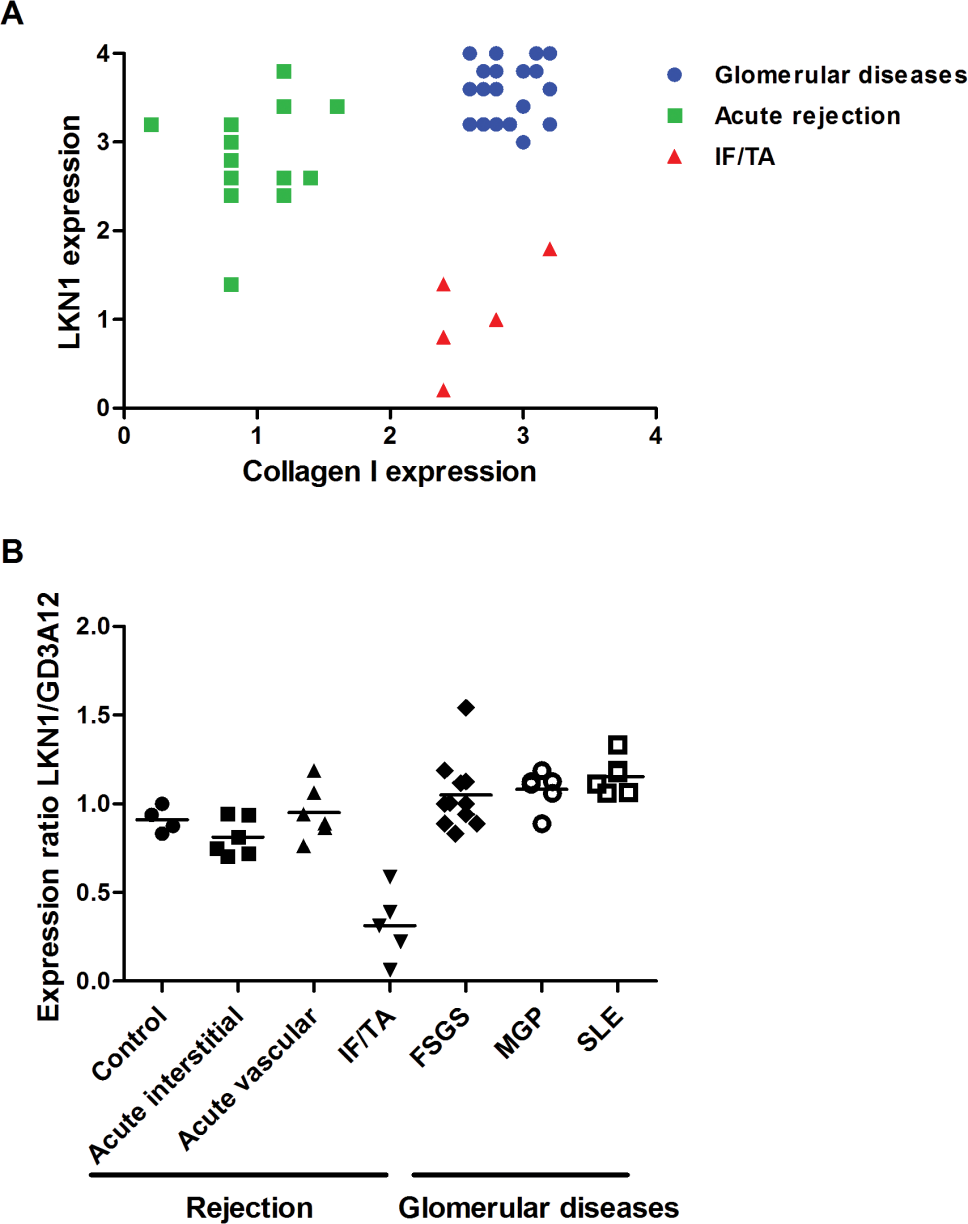
Correlation between the expression of the 4/2,4-di-O-sulfated DS domain recognized by antibody LKN1 and type I collagen (A) and the expression ratio of LKN1 and GD3A12 (B) in glomerular diseases, acute rejection and IF/TA. In acute (vascular and interstitial) rejection, the interstitial expression of the 4/2,4-di-O-sulfated DS domain recognized by antibody LKN1 is increased, while expression of collagen type I is decreased (A). Expression of collagen type I is increased in glomerular diseases and IF/TA, which is accompanied by an increased expression of the 4/2,4-di-O-sulfated DS domain in glomerular diseases and a decreased expression of the 4/2,4-di-O-sulfated DS domain in IF/TA. The expression ratio of the 4/2,4-di-O-sulfated DS domain recognized by antibody LKN1 to the IdoA-Gal-NAc4S DS domain recognized by antibody GD3A12 is decreased in the IF/TA patients in contrast to the patients with acute rejection or glomerular diseases (B).

In summary, we observed a differential expression of the 4/2,4-di-O-sulfated DS domain, recognized by antibody LKN1, in renal transplant rejection and glomerular pathologies. We analyzed the correlation between the expression of 4/2,4-di-O-sulfated DS domain recognized by antibody LKN1 and type I collagen in glomerular diseases, acute rejection and IF/TA, which may clarify the difference of LKN1 staining in the different renal diseases (Fig 5A). We observed 3 patterns; 1) in acute allograft rejection expression of the 4/2,4-di-O-sulfated DS domain recognized by LKN1 is increased more than collagen type I; 2) in glomerular disease both expressions are increased; 3) but in IF/TA expression of the 4/2,4-di-O-sulfated DS domain is decreased, while collagen type I is increased. In contrast to the differential interstitial expression of the 4/2,4-di-O-sulfated DS domain recognized by antibody LKN1 among the various renal diseases, the expression of the IdoA-Gal-NAc4S DS domain recognized by GD3A12 is similar in healthy and diseased kidneys. Therefore, the expression ratio of 4/2,4-di-O-sulfated DS domain to IdoA-Gal-NAc4S DS domain might be useful in monitoring the progression of IF/TA (Fig 5B). This information may be useful for diagnostic purposes on renal biopsies.

## Discussion

This study shows the differential expression of the specific 4/2,4-di-O-sulfated and IdoA-Gal-NAc4S DS domains recognized by the anti-DS antibodies LKN1 and GD3A12, respectively, in normal kidney and in renal pathology. Previous studies showed the expression of DS (and CS) in the kidney, both in the glomerulus and in the tubular interstitium [14, 24, 25]. The 4/2,4-di-O-sulfated DS domain is expressed in the tubular interstitial space, but not in the healthy glomerulus, as is type I collagen [20]. Furthermore, the normal expression pattern of the 4/2,4-di-O-sulfated DS domain resembles the expression of the two DSPGs, decorin and biglycan, which are also expressed in the renal tubular interstitium with accumulations around the tubules, but no expression in the normal glomerulus [15, 26]. For the IdoA-Gal-NAc4S DS domain, the expression pattern in control rat kidney was previously shown in large blood vessels with a weaker staining present in the tubular interstitial space and near Bowman’s capsule [21]. DS and DSPGs have been reported to be increased in several fibrotic renal diseases, including interstitial fibrosis, diabetic nephropathy, mesangial sclerosis and nephrosclerosis [14, 15]. In the unilateral urethral obstruction (UUO) model in the rat, a well established model of renal inflammation and fibrosis, fibrotic lesions showed ten-fold increase in CS/DS content and a decrease in the sulfation degree of CS/DS, accompanied by a lower IdoA content [14].

The expression of the 4/2,4-di-O-sulfated DS domain recognized by LKN1 was significantly decreased in the tubular interstitium in biopsies of patients with IF/TA compared to biopsies of patients with acute rejection. In IF/TA there was an inverse correlation between the decreased expression of the 4/2,4-di-O-sulfated DS domain and the increased expression of decorin, TGF-β and type I collagen. Importantly, glomerular LKN1 staining was not observed in controls, while patients with different forms of renal allograft rejections did show a glomerular LKN1 staining. The increased glomerular expression of the 4/2,4-di-O-sulfated DS domain correlated with the increased glomerular expression of TGF-β in the biopsies with acute rejection and IF/TA, which corresponds to other studies [27, 28]. In biopsies of patients with glomerular disease, there was also an increased glomerular expression of the 4/2,4-di-O-sulfated DS domain, which was accompanied by glomerular expression of type I collagen and TGF-β. These data are in accordance to other studies that showed an upregulated expression of small leucine-rich proteoglycans and type I collagen in renal fibrosis, diabetic nephropathy and crescentic glomerulonephritis [29–31]. The expression of the 4/2,4-di-O-sulfated DS domain was significantly increased in the tubular interstitium of patients with glomerular disease, which was again accompanied by an increased expression of type I collagen, suggesting a role for this 4/2,4-di-O-sulfated DS domain in matrix remodelling and collagen formation in glomerular disease. In patients with FSGS and SLE, the increased expression of the 4/2,4-di-O-sulfated DS domain in the tubular interstitium, correlated with the increased expression of TGF-β, which was in accordance with the degree of interstitial fibrosis and tubular atrophy. This was also shown by other studies [32, 33]. Since the specific 4/2,4-di-O-sulfated DS domain is present at an earlier stage in the analyzed nephropathies, this anti-DS antibody might be useful in determining early changes in matrix deposition in the diseased kidney. It has been described that DS size increases in response to an insult, but returns to normal later on, thus regulating collagen fibril formation [34]. The increase in DS staining observed in the tubular interstitium using our anti-DS antibody LKN1 might reflect the changes in DS size and sulfation needed for initial matrix modelling. Type I collagen depositions were present in the analyzed glomerular diseases and there was a positive correlation with the expression of the DSPG decorin, TGF-β and the specific 4/2,4-di-O-sulfated DS domain recognized by LKN1, which indicates that the specific 4/2,4-di-O-sulfated DS domain plays different pathophysiological roles in renal allograft rejection and glomerular diseases. In IF/TA intima fibrosis/hyperplasia leads to arteriolopathy, which can induce ischemia. It is tempting to speculate that ischemia may induce a decreased expression of the specific 4/2,4-di-O-sulfated DS domain in contrast to the increased expression in glomerular diseases with less or no ischemia. Another study [35] showed that increased 6-*O*-sulfation of HS regulates the fibrotic response associated with chronic renal allograft failure. Interestingly, TGF-β induced up regulation of HS-6-*O*-endosulfatase-2 expression, which led to this changed sulfation pattern. In our study, the expression of the sulfotransferases dermatan 4-*O*-sulfotransferase 1 and /or the CS/DS-2-O-sulfotransferase may be changed in the glomeruli and tubular interstitium during the different stages of renal allograft rejection and glomerular diseases, causing differential expression of specific sulfated DS domains.

In contrast to the differential expression of the 4/2,4-di-O-sulfated DS domain in the control, the different types of renal allograft rejections and the glomerular diseases, the expression of the IdoA-Gal-NAc4S DS domain recognized by the anti-DS antibody GD3A12 was not different for either the tubular interstitium or the glomerulus. Therefore, these data indicate a dynamic role for the specific DS domains defined by the anti-DS antibody LKN1 in renal diseases. Moreover, it has been shown that GAGs are involved in chemokine sequestration in renal allograft rejection, allowing an inflammatory reaction to take place which can ultimately lead to transplant rejection [36]. An increase in CS expression was seen during acute renal allograft rejection and the biopsy specimens showed an enhanced capacity of binding a specific chemokine (CCL5). In a recent study using an experimental renal transplantation model an up regulation of the CSPG versican in kidneys with increased interstitial fibrosis was shown [10].

In summary, we observed a differential expression of DS domains when comparing healthy kidneys and diseased kidneys. Our data suggest a role for the 4/2,4-di-O-sulfated DS domain recognized by antibody LKN1 in renal diseases with early fibrosis. However, further research is required to delineate the exact role of different DS domains in renal fibrosis. The described anti-DS antibodies might contribute to the future development of glycomimetics, which may ultimately lead to a better treatment of selected renal nephropathies and prevention of renal rejection.

## References

[1] Malavaki C, Mizumoto S, Karamanos N, Sugahara K. Recent advances in the structural study of functional chondroitin sulfate and dermatan sulfate in health and disease. Connective tissue research. 2008;49(3):133–9. 10.1080/03008200802148546 18661328

[2] Trowbridge JM, Gallo RL. Dermatan sulfate: new functions from an old glycosaminoglycan. Glycobiology. 2002;12(9):117R–25R. 12213784 10.1093/glycob/cwf066

[3] Sugahara K, Mikami T, Uyama T, Mizuguchi S, Nomura K, Kitagawa H. Recent advances in the structural biology of chondroitin sulfate and dermatan sulfate. Current opinion in structural biology. 2003;13(5):612–20. 14568617 10.1016/j.sbi.2003.09.011

[4] Corsi A, Xu T, Chen XD, Boyde A, Liang J, Mankani M, et al Phenotypic effects of biglycan deficiency are linked to collagen fibril abnormalities, are synergized by decorin deficiency, and mimic Ehlers-Danlos-like changes in bone and other connective tissues. Journal of bone and mineral research: the official journal of the American Society for Bone and Mineral Research. 2002;17(7):1180–9. 10.1359/jbmr.2002.17.7.1180 12102052

[5] Danielson KG, Baribault H, Holmes DF, Graham H, Kadler KE, Iozzo RV. Targeted disruption of decorin leads to abnormal collagen fibril morphology and skin fragility. The Journal of cell biology. 1997;136(3):729–43. 9024701 10.1083/jcb.136.3.729 PMC2134287

[6] Danielsen CC. Mechanical properties of reconstituted collagen fibrils. Influence of a glycosaminoglycan: dermatan sulfate. Connective tissue research. 1982;9(4):219–25. 6215205 10.3109/03008208209160265

[7] Schaefer L, Macakova K, Raslik I, Micegova M, Grone HJ, Schonherr E, et al Absence of decorin adversely influences tubulointerstitial fibrosis of the obstructed kidney by enhanced apoptosis and increased inflammatory reaction. The American journal of pathology. 2002;160(3):1181–91. 11891213 10.1016/S0002-9440(10)64937-1 PMC1867182

[8] Chapman JR, O'Connell PJ, Nankivell BJ. Chronic renal allograft dysfunction. Journal of the American Society of Nephrology: JASN. 2005;16(10):3015–26. 16120819 10.1681/ASN.2005050463

[9] El-Zoghby ZM, Stegall MD, Lager DJ, Kremers WK, Amer H, Gloor JM, et al Identifying specific causes of kidney allograft loss. American journal of transplantation: official journal of the American Society of Transplantation and the American Society of Transplant Surgeons. 2009;9(3):527–35. 10.1111/j.1600-6143.2008.02519.x 19191769

[10] Rienstra H, Katta K, Celie JW, van Goor H, Navis G, van den Born J, et al Differential expression of proteoglycans in tissue remodeling and lymphangiogenesis after experimental renal transplantation in rats. PloS one. 2010;5(2):e9095 10.1371/journal.pone.0009095 20140097 PMC2816722

[11] Papaioannou EG, Magkou C, Kostakis A, Staikou C, Mitsoula P, Kyriakides S, et al Changes in total serum glycosaminoglycan levels in patients undergoing renal transplantation: preliminary data. Surgery today. 2004;34(8):668–72. 15290396 10.1007/s00595-004-2795-4

[12] Deng S, Pascual M, Lou J, Buhler L, Wessel HP, Grau G, et al New synthetic sulfated oligosaccharides prolong survival of cardiac xenografts by inhibiting release of heparan sulfate from endothelial cells. Transplantation. 1996;61(9):1300–5. 8629287 10.1097/00007890-199605150-00003

[13] Stevens RB, Wang YL, Kaji H, Lloveras J, Dalmasso A, Bach FH, et al Administration of nonanticoagulant heparin inhibits the loss of glycosaminoglycans from xenogeneic cardiac grafts and prolongs graft survival. Transplantation proceedings. 1993;25(1 Pt 1):382 8438344

[14] Koshiishi I, Hasegawa T, Imanari T. Quantitative and qualitative alterations of chondroitin/dermatan sulfates accompanied with development of tubulointerstitial nephritis. Archives of biochemistry and biophysics. 2002;401(1):38–43. 12054485 10.1016/S0003-9861(02)00032-2

[15] Stokes MB, Holler S, Cui Y, Hudkins KL, Eitner F, Fogo A, et al Expression of decorin, biglycan, and collagen type I in human renal fibrosing disease. Kidney international. 2000;57(2):487–98. 10652025 10.1046/j.1523-1755.2000.00868.x

[16] Hadad SJ, Michelacci YM, Schor N. Proteoglycans and glycosaminoglycans synthesized in vitro by mesangial cells from normal and diabetic rats. Biochimica et biophysica acta. 1996;1290(1):18–28. 8645702 10.1016/0304-4165(95)00183-2

[17] Pecly IM, Goncalves RG, Rangel EP, Takiya CM, Taboada FS, Martinusso CA, et al Effects of low molecular weight heparin in obstructed kidneys: decrease of collagen, fibronectin and TGF-beta, and increase of chondroitin/dermatan sulfate proteoglycans and macrophage infiltration. Nephrology, dialysis, transplantation: official publication of the European Dialysis and Transplant Association—European Renal Association. 2006;21(5):1212–22. 10.1093/ndt/gfk076 16421158

[18] Reine TM, Grondahl F, Jenssen TG, Hadler-Olsen E, Prydz K, Kolset SO. Reduced sulfation of chondroitin sulfate but not heparan sulfate in kidneys of diabetic db/db mice. The journal of histochemistry and cytochemistry: official journal of the Histochemistry Society. 2013;61(8):606–16. 23757342 10.1369/0022155413494392 PMC3724391

[19] Sorrell JM, Carrino DA, Baber MA, Asselineau D, Caplan AI. A monoclonal antibody which recognizes a glycosaminoglycan epitope in both dermatan sulfate and chondroitin sulfate proteoglycans of human skin. The Histochemical journal. 1999;31(8):549–58. 10507462 10.1023/a:1003896124595

[20] Lensen JF, Wijnhoven TJ, Kuik LH, Versteeg EM, Hafmans T, Rops AL, et al Selection and characterization of a unique phage display-derived antibody against dermatan sulfate. Matrix biology: journal of the International Society for Matrix Biology. 2006;25(7):457–61. 16934446 10.1016/j.matbio.2006.06.003

[21] Ten Dam GB, Yamada S, Kobayashi F, Purushothaman A, van de Westerlo EM, Bulten J, et al Dermatan sulfate domains defined by the novel antibody GD3A12, in normal tissues and ovarian adenocarcinomas. Histochemistry and cell biology. 2009;132(1):117–27. 10.1007/s00418-009-0592-2 19360434

[22] Racusen LC, Solez K, Colvin RB, Bonsib SM, Castro MC, Cavallo T, et al The Banff 97 working classification of renal allograft pathology. Kidney international. 1999;55(2):713–23. 9987096 10.1046/j.1523-1755.1999.00299.x

[23] Solez K, Colvin RB, Racusen LC, Sis B, Halloran PF, Birk PE, et al Banff '05 Meeting Report: differential diagnosis of chronic allograft injury and elimination of chronic allograft nephropathy ('CAN'). American journal of transplantation: official journal of the American Society of Transplantation and the American Society of Transplant Surgeons. 2007;7(3):518–26. 10.1111/j.1600-6143.2006.01688.x 17352710

[24] Klein DJ, Brown DM, Oegema TR Jr., Partial characterization of heparan and dermatan sulfate proteoglycans synthesized by normal rat glomeruli. The Journal of biological chemistry. 1986;261(35):16636–52. 2946688

[25] van de Lest CH, Versteeg EM, Veerkamp JH, van Kuppevelt TH. Quantification and characterization of glycosaminoglycans at the nanogram level by a combined azure A-silver staining in agarose gels. Analytical biochemistry. 1994;221(2):356–61. 7529008 10.1006/abio.1994.1425

[26] Schaefer L, Grone HJ, Raslik I, Robenek H, Ugorcakova J, Budny S, et al Small proteoglycans of normal adult human kidney: distinct expression patterns of decorin, biglycan, fibromodulin, and lumican. Kidney international. 2000;58(4):1557–68. 11012890 10.1046/j.1523-1755.2000.00317.x

[27] Baboolal K, Jones GA, Janezic A, Griffiths DR, Jurewicz WA. Molecular and structural consequences of early renal allograft injury. Kidney international. 2002;61(2):686–96. 11849412 10.1046/j.1523-1755.2002.00149.x

[28] Jain S, Furness PN, Nicholson ML. The role of transforming growth factor beta in chronic renal allograft nephropathy. Transplantation. 2000;69(9):1759–66. 10830203 10.1097/00007890-200005150-00001

[29] Schaefer L. Small leucine-rich proteoglycans in kidney disease. Journal of the American Society of Nephrology: JASN. 2011;22(7):1200–7. 10.1681/ASN.2010050570 21719787

[30] Schaefer L, Raslik I, Grone HJ, Schonherr E, Macakova K, Ugorcakova J, et al Small proteoglycans in human diabetic nephropathy: discrepancy between glomerular expression and protein accumulation of decorin, biglycan, lumican, and fibromodulin. FASEB journal: official publication of the Federation of American Societies for Experimental Biology. 2001;15(3):559–61. 11259366 10.1096/fj.00-0493fje

[31] Stokes MB, Hudkins KL, Zaharia V, Taneda S, Alpers CE. Up-regulation of extracellular matrix proteoglycans and collagen type I in human crescentic glomerulonephritis. Kidney international. 2001;59(2):532–42. 11168935 10.1046/j.1523-1755.2001.059002532.x

[32] Onetti Muda A, Feriozzi S, Rahimi S, Faraggiana T. Expression of TGF-beta receptors type I and II in human glomerulonephritis. Nephrology, dialysis, transplantation: official publication of the European Dialysis and Transplant Association—European Renal Association. 1998;13(2):279–84. 10.1093/oxfordjournals.ndt.a027819 9509435

[33] Yang CW, Hsueh S, Wu MS, Lai PC, Huang JY, Wu CH, et al Glomerular transforming growth factor-beta1 mRNA as a marker of glomerulosclerosis-application in renal biopsies. Nephron. 1997;77(3):290–7. 9375822 10.1159/000190290

[34] Kuwaba K, Kobayashi M, Nomura Y, Irie S, Koyama Y. Size control of decorin dermatan sulfate during remodeling of collagen fibrils in healing skin. Journal of dermatological science. 2002;29(3):185–94. 12234708 10.1016/s0923-1811(02)00023-3

[35] Alhasan AA, Spielhofer J, Kusche-Gullberg M, Kirby JA, Ali S. Role of 6-O-sulfated heparan sulfate in chronic renal fibrosis. The Journal of biological chemistry. 2014;289(29):20295–306. 10.1074/jbc.M114.554691 24878958 PMC4106343

[36] Ali S, Malik G, Burns A, Robertson H, Kirby JA. Renal transplantation: examination of the regulation of chemokine binding during acute rejection. Transplantation. 2005;79(6):672–9. 15785373 10.1097/01.tp.0000155961.57664.db

